# Trans-arterial embolization for treatment of acute lower gastrointestinal bleeding—a multicenter analysis

**DOI:** 10.1007/s00330-024-11102-x

**Published:** 2024-10-16

**Authors:** Clarissa Hosse, Maximilian Moos, Lena S. Becker, Malte Sieren, Lukas Müller, Fabian Stoehr, Benedikt M. Schaarschmidt, Gianluca Barbone, Federico Collettini, Uli Fehrenbach, Jan B. Hinrichs, Roman Kloeckner, Dominik Geisel, Frank Tacke, Bernhard Gebauer, Timo A. Auer

**Affiliations:** 1 Department of Radiology, Charité – Universitätsmedizin Berlin, Corporate Member of Freie Universität and Humboldt-Universität zu Berlin, 10117 Berlin, Germany; 2 Department of Diagnostic and Interventional Radiology, University Medical Center Mainz, Mainz, Germany; 3 Institute of Diagnostic and Interventional Radiology, Hannover Medical School, 30625 Hannover, Germany; 4 Institute of Interventional Radiology, University Hospital Schleswig-Holstein-Campus Lübeck, Lübeck, Germany; 5 Institute of Diagnostic and Interventional Radiology and Institute for Artificial Intelligence in Medicine, University Hospital Essen, Essen, Germany; 6 Department of Hepatology and Gastroenterology, Charité – Universitätsmedizin Berlin, Campus Virchow-Klinikum (CVK) and Campus Charité Mitte (CCM), 13353 Berlin, Germany; 7 Berlin Insitute of Health at Charité-Universitätsmedizin Berlin, 10117 Berlin, Germany; 8 Clinic for Diagnostic and Interventional Radiology and Neuroradiology, St. Bernward Krankenhaus Hildesheim, Hildesheim, Germany

**Keywords:** Trans-arterial embolization (TAE), Lower gastrointestinal bleeding (LGIB), Embolization

## Abstract

**Purpose:**

To assess the technical feasibility, safety, and clinical success rate of trans-arterial embolization (TAE) as an emergency treatment for acute lower gastrointestinal bleeding (LGIB).

**Materials and methods:**

Consecutive patients who received urgent TAE due to active LGIB at five academic centers in Germany were retrospectively analyzed. LGIB was confirmed and localized using contrast-enhanced computed tomography (CT) or endoscopy. Outcome parameters including technical and clinical success rates as well as ischemia-related adverse events were analyzed. Furthermore, treatment-related variables that may affect technical and clinical success were analyzed using a regression model.

**Results:**

One hundred and forty-one patients were included. TAE was performed in 91% (128/141) of patients. In 81% (114/141) of patients, TAE was performed due to active bleeding visible at angiography, the remaining 10% (14/141) underwent empiric embolization based on pre-interventional imaging. In 9% (13/141) of patients, no TAE was performed. Microcoils were the most used embolic 48.5% (62/128), followed by glue 23.5% (30/128) and Microparticles (8%; 10/128). In the case of bleeding visible in angiography, the technical success rate was 100% (114/114); the clinical success rate was 93.6% (120/128). Severe ischemia-related adverse events necessitating bowel surgery occurred in 14% (18/128) of all patients after embolization. Thirty-day mortality was 14% (21/141). Regression analysis revealed no significant correlations but a statistical trend toward a higher incidence of bowel resection when glue was used (*p* = 0.090) and toward a higher 30-day mortality when an unselective embolization was performed (*p* = 0.057).

**Conclusion:**

TAE for LGIB has a high technical and clinical success rate. Severe ischemia-related adverse events necessitating bowel surgery occurred in 14% of patients without identifying a significant correlation to the embolization technique or an embolic.

**Key Points:**

***Question*** *Is trans-arterial embolization (TAE) viable as an emergency treatment for acute lower gastrointestinal bleeding (LGIB)?*

***Findings*** *TAE demonstrated a 100% technical and 93.6% clinical success rate in treating acute LGIB, with severe ischemia-related adverse events occurring in 14% of patients.*

***Clinical relevance*** *TAE is highly effective and has an acceptable complication rate in treating lower gastrointestinal bleeding, emphasizing the need for a direct head-to-head comparison between endovascular and endoscopic therapy*.

## Introduction

Lower gastrointestinal bleeding (LGIB) accounts for about 20% of acute gastrointestinal (GI) bleeding events with an estimated incidence of 33–87/100,000 and is defined as bleeding originating from a source located beyond the duodenum-jejunal junction, encompassing the small intestine, colon, rectum, and anus [1, 2]. Although LGIB tends to resolve on its own and manifests with less severe clinical symptoms than upper gastrointestinal bleeding (UGIB), mortality is strongly related to comorbidities, ranging from 3 to 18% [2, 3]. Current guidelines recommend that hemodynamically unstable patients with suspected ongoing LGIB undergo emergency computed tomography angiography to locate the site of bleeding, followed by endoscopic or radiologic treatment [4, 5]. Selective trans-arterial embolization (TAE) has emerged as an effective and well-tolerated therapeutic option for patients with LGIB, and the current literature emphasizes its safety [6]. However, most published data have been obtained in relatively small, single-center patient populations [1].

**Fig. 1:**
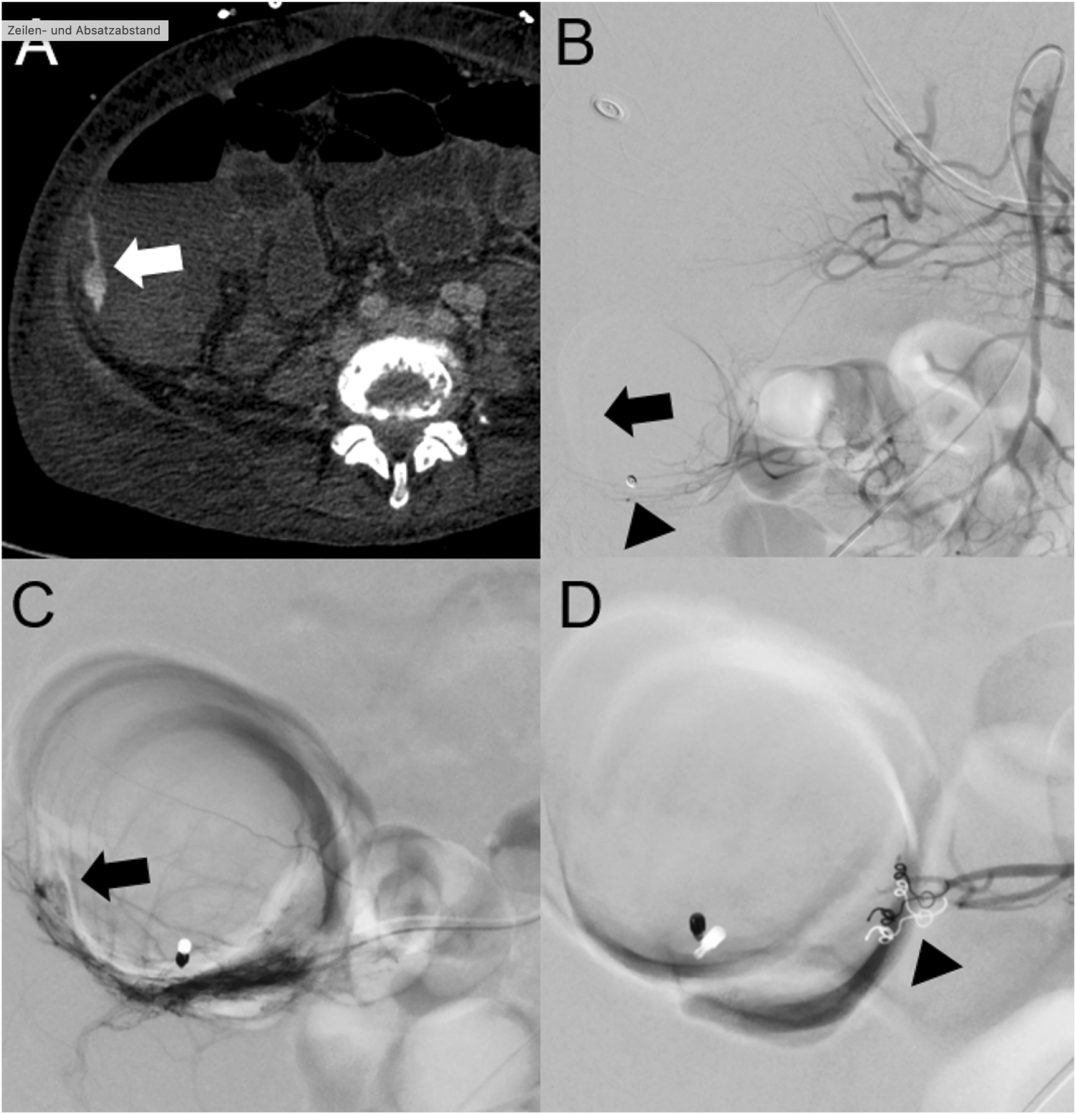
**A** A 62-year-old female patient with liver cirrhosis and active bleeding at the cecal pole with a clear extravasation (white arrow) on angio-CT. **B** Digital subtraction angiography showing a mesenteric image. The black arrow is pointing at the region of bleeding referring to the angio-CT (Black arrowhead: endoscopic clip). **C** Selective angiography with an extravasation. **D** Technically successful selective embolization with particles (Contour 355-510) and Coils (black arrowhead)

TAE is a safe treatment option for patients with abdominal bleeding and is used for a variety of indications. However, in LGIB, endoscopic treatment remains the preferred method in clinical practice, probably due to its historical precedence and better availability [1, 7]. Nevertheless, endoscopic procedures often fail due to inadequate patient preparation and extensive bleeding. In addition, there are localizations in the small intestine where endoscopy is extremely limited for technical reasons [1, 8–11]. In view of both the efficacy and safety of endovascular interventions and the constraints associated with endoscopic therapies, some guidelines regard both approaches as equally viable options [2]. The British Society of Gastroenterology, for example, recommends either interventional radiology or endoscopic treatment for patients classified as unstable and with extravasation seen on computed tomography angiography (CTA) (Fig. 1). As there is no direct head-to-head comparison between endovascular therapy and endoscopy, the choice of treatment should be based on individual patient factors, local expertise, and resource availability [2]. As TAE is increasingly recommended and accordingly in demand, more data are needed to guide treatment-choice. We therefore conducted a multicentric study to investigate technical and clinical success rates, as well as ischemic-related adverse events, in a large population of patients who underwent TAE for LGIB at five university hospitals in Germany.

## Materials and methods

### Patients

For this Institutional Review Board-approved retrospective study, we collected data from consecutive patients with LGIB who underwent angiography for embolization at five academic centers in Germany between January 2010 and December 2023 (EA4/087/23). LGIB was defined as arterial hemorrhage occurring distal to the duodenal-jejunal flexure. A total of 141 angiographies were performed. All written records on LGIB angiograms were analyzed for instances of angiographic anomalies and embolization attempts. No patients were excluded. Outcome was analyzed in terms of clinical success and adverse events. Patient demographics of the study population are summarized in Table 1, and the procedural details are summarized in Table 2. Use of vasopressors, antiplatelets, and therapeutically dosed anticoagulant medications during the 48 h prior to embolization treatment was evaluated. Patients with a systolic blood pressure reading of less than 90 mm Hg in the 48 h before the intervention were classified as hemodynamically unstable. LGIB was localized using contrast-enhanced computed tomography (CT) and endoscopy. In 36.9% (52/141), endoscopy was performed prior to CT.

**Table 1:** Patient demographics and pretreatment characteristics

Characteristic			
Male sex/female sex	Male: 69.5% (98); female: 30.5% (43)		
Age, years (median)	64 (ICR; 54.0–75.0)		
Comorbidities			
End-stage renal disease	27.0% (38)		
Cardiovascular disease	17.5% (25)		
Prior gastrointestinal surgery	13.5% (19)		
Gastrointestinal disease	12.0% (17)		
Cirrhosis	5.0% (7)		
Coagulation disorder	4.5% (6)		
Malignancy	2.0% (3)		
Pulmonary disease	7.0% (10)		
None	11.5% (16)		
Site of hemorrhage			
Middle GI tract	67.0% (95/141)		
Lower GI tract	31.5% (44/141)		
Both	1.5% (2/141)		
Anticoagulation			
No	62% (87)		
Yes	38% (54)		
ASS	11.0% (15)		
Heparin	3% (4)		
DPA	7% (10)		
ASS + NMH	1.5% (2)		
DOAC	3.5% (5)		
Phenprocoumon	2.0% (3)		
NOS	6.5% (9)		
Unknown	3.0% (4)		
**Laboratory data**	**Baseline**	**+** **14 days**	***p*-values**
Lactate mg/dL (*n* = 87)	2.6 (1.1–10.0)	1.9 (1.0–7.0)	< 0.0001
Leukocytes /nL (*n* = 115)	12.7 (9.1–17.9)	8.6 (6.2–13.0	< 0.0001
C-reactive protein mg/L (*n* = 114)	46.5 (18.3–105.8)	52.7 (16.9–136.3)	< 0.0001
Procalcitonin ng/L (*n* = 56)	0.8 (0.4–3.5)	0.3 (0.2–1.5)	< 0.0001
Pretreatment			
Transfusion	13% (18)		
Endoscopy	38% (52)		
Angiography	8.5% (12)		
Surgery	39% (53)		
None	1.5% (2)		
Clinical condition			
Stable	49% (69)		
Stable with blood transfusion	26% (37)		
Unstable	25% (35)		

*ASS* acetylsalicylic acid, *DPA* dual platelet aggregation, *LMWH* low molecular weight heparin, *DOAC* direct oral anticoagulants, *NOS* non-other specified

**Table 2:** Procedure-related outcomes and technical and clinical information

Technical and clinical results	
Bleeding visible during procedure	81.0% (114/141)
Technical success	100.0% (114/114)
Endovascular embolization	91.0% (128/141)
Empirical embolization	10.0% (14/141)
Primary clinical success	93.8% (120/128)
Ischemia-related bowel surgery	14.0% (18/128)
30-day mortality	14.0% (21/141)
Embolic agents and devices	
Microcoils	48.5% (62)
Glue	23.5% (30)
Microcoils + glue	9.5% (12)
Particles	8.0% (10)
Microcoils + particles	7.0% (9)
Stent graft*	1.5% (2)
Plug	< 1% (1)
Microcoils + glue + particles	< 1% (1)
Microcoils + stent*	< 1% (1)
Embolization position	
Superselective	61% (78/128)
Selective	30.5% (39/128)
Semiselective	8.5% (11/128)
Unselective	0% (0/128)

* Stentgrafts are, per definition, no embolic agents or devices

### Angiography and embolization technique

The right common femoral artery was used for ultrasound-guided access under local anesthesia. Angiography was performed with moderate sedation. A 5-F vascular access sheath was inserted into the right common femoral artery. Angiography of the superior or inferior mesenteric arteries or the rectal arteries was performed. For superselective angiography, a microcatheter was always used once contrast extravasation was detected. For embolization, the microcatheter was advanced as far distally as feasible. Various embolic materials and devices, including microcoils, particles, glue, stent graft (per definition, no embolic agent or device), or plugs, were used alone or in different combinations (Table 2, Figs. S1 and S2). Product names of all devices at each center are added in Supplementary information (S1). A total of 128 embolizations were performed. Microcoils were the most commonly used embolic 48.5% (62/128), followed by glue 23.5% (30/128) and Microparticles (8%; 10/128). In the remaining cases, various combinations of embolics were used (Table 2).

### Technical and clinical outcomes

All procedural reports, clinical notes, imaging findings, endoscopy reports, and surgical notes were reviewed for assessing embolization outcomes. We included 141 procedures in patients presenting with clinical signs of LGIB. Technical success was defined as the immediate endovascular termination of bleeding by embolization seen as the absence of extravasation on angiography (Fig. 1). Clinical success was defined as the absence of clinical bleeding symptoms in the lower GI tract within 30 days after angiographic embolization assessed by clinical and imaging follow-up [12]. An embolization was defined as empirical if the bleeding was visible on the CT scan but no longer during angiography, yet was still performed based on the anatomical correlation. The occurrence of bleeding symptoms within 30 days of embolization was diagnosed as recurrent bleeding. Re-therapies (angiographic, surgical, endoscopic, or other) were also recorded. Angiography reports and images were compared with pathology findings for resected bowel. If the site of embolization corresponded to the segment of bowel resected, findings in resected specimens were used to confirm or rule out ischemic changes.

### Statistical analysis

All statistical analyses were performed using XLSTAT statistical and data analysis solution (Addinsoft), IBM SPSS Statistics for Windows, version 28.0 (IBM Corp.), and Jamovi Version 2.3 (The jamovi project). Descriptive statistics were carried out for all variables. Binominal single-variate and multivariate logistic regression was used to analyze the influence of categorical variables on clinical success or complications. A *p*-value of less than 0.05 was considered statistically significant.

## Results

### Demographic data

The study population consisted of 141 patients (male: 69.5% (*n* = 98); female: 30.5% (*n* = 43)) with a median age of 64 years (ICR; 54.0–75.0). Overall, 88.5% of patients (125/141) presented with significant pre-existing conditions regarding the occurrence and course of gastrointestinal bleeding. The most common conditions were prior gastrointestinal surgery (13.5%), gastrointestinal diseases (including gastroesophageal reflux disease, peptic ulcers, inflammatory bowel disease, irritable bowel syndrome, celiac disease, gallstones, liver diseases, pancreatitis, diverticular disease, gastroenteritis, and hemorrhoids) (12.0%), and coagulation disorders (including inherited conditions like hemophilia and von Willebrand disease, acquired conditions like deep vein thrombosis and disseminated intravascular coagulation, and platelet disorders like thrombocytopenia) (4.5%) (Table 1).

### Technical and clinical results

**Fig. 2:**
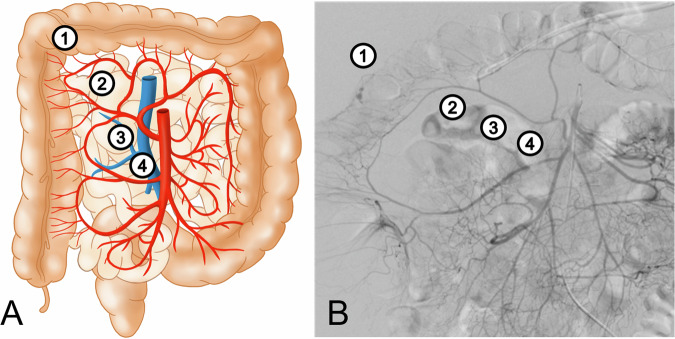
**A** Own schematic drawing to illustrate the mesenteric anatomy and the embolization positions. **B** Digital subtraction angiography showing a mesenteric imagesuperior mesenteric angiography—embolization positions 1–4 using the colon as an example: (1) Superselective embolization position from a terminal arterial arcade originating from a segmental vascular branch and usually originating from a branch of the third order. (2) Selective embolization position from a segmental vascular branch (third order), which originates from one of the main branches of the superior mesenteric artery (middle colic artery f.e.). (3) Semiselective embolization position from a distal position of one of the main branches of the superior mesenteric artery (second order). (4) Unselective embolization position from a proximal position of one of the main branches of the superior mesenteric artery (second order) or the superior mesenteric artery itself (first order)

All patients showed signs of active bleeding in pre-interventional imaging. In 67.0% (95/141), the bleeding site was located in the mid-GI tract between the duodeno-jejunal flexure and the ileocaecal valve. In 31.5% (44/141), it was located in the lower GI tract between the ileocaecal valve and the rectum, and in 1.5% (2/141), bleeding foci were seen in both the middle and lower GI tract (Table 1). In 81% (114/141) of cases, the target of embolization was visible, while a pseudoaneurysm was detected in 5.3% (6/114). In these patients, technical success was 100% (114/114). In 10% (14/141), an empirical embolization was performed. A total of 91% (128/141) had an endovascular embolization. The primary clinical success rate was 93.8% (120/128). Regarding the embolization position, a superselective position was achieved in 61% (78/128) of cases, while in 30.5% (39/128) a selective and in 8.5% (11/128) a semiselective position was achieved. No unselective embolization was performed in any of the study patients (Fig. 2 and Table 2). For the distribution of the embolic agents and devices used, please see the “Angiography and embolization technique” section in “Materials and methods” and Table 2. Fifty-one percent of study patients were classified as either clinically unstable or stable while receiving blood transfusion. Severe ischemia-related adverse events after embolization necessitating bowel surgery occurred in 14.0% (18/128) of all patients. Total 30-day mortality was 14.0% (21/141) (Table 2).

### Determinants of clinical and technical success

The embolic materials and devices used did neither correlate significantly with clinical success (*p* = 0.509–0.999) nor the need for bowel resection due to ischemia (*p* = 0.090–0.996), though there was a statistical trend toward a higher incidence of bowel resection when glue was used (*p* = 0.090). Selectivity did not show a significant correlation with clinical success (*p* = 0.994–0.995) or the need for bowel resection either (*p* = 0.166–0.575). Thirty-day mortality showed no significant correlation with the type of embolic material (*p* = 0.131–0.994) or selectivity (*p* = 0.057–0.869), although there was a statistical trend toward an unselective embolization position (*p* = 0.057). Other factors such as age, sex, comorbidities, and bleeding localization also showed no significant correlation (*p* > 0.05).

## Discussion

Overall, our results confirm the high safety and effectiveness of TAE in treating LGIB in a multicenter setting. In cases with bleeding visible on endovascular angiography, we observed a technical success rate of 100% and a clinical success rate of 93.8% with a severe ischemia-related adverse event rate of 14.0%.

In our study population, embolization was performed in 91% of the patients who were scheduled for therapeutic angiography. However, angiography revealed evidence of bleeding in only 81% of cases, indicating that empirical embolization was administered in 14 of the 128 patients who underwent the procedure. The high embolization rate (91%) underlines the effective diagnostic management of patients presenting with clinically suspected LGIB, given that CTA was routinely conducted prior to endovascular angiography at all participating centers. Performing CTA for suspected LGIB is recommended even prior to therapeutic angiography, as it provides valuable information on the origin and intensity of bleeding, its location, and vascular anatomy [13, 14]. Empirical embolization, for example, can only be performed when a CTA map of the individual patient’s vascular anatomy is available. CTA detects Gl bleeding with a flow rate of at least 0.3 mL/min with a sensitivity ranging from 50 to 86% and a specificity of 92 to 95%. In some studies, sensitivity rates of up to 100% and specificities of 96% have been reported [14, 15]. Diagnostic angiography can identify the source of bleeding when the bleeding rate ranges from 0.5 to 1 mL/min, depending on the selectivity of contrast agent injection. Moreover, bleeding may have been stopped or is masked by the intensive care measures taken in the interval between CTA and angiography [14, 16]. Another highly sensitive method for detecting gastrointestinal bleeding is red blood count scintigraphy. However, this nuclear medicine technique, which tracks the movement of red blood cells, is an imaging modality that is not widely accessible [17].

**Fig. 3:**
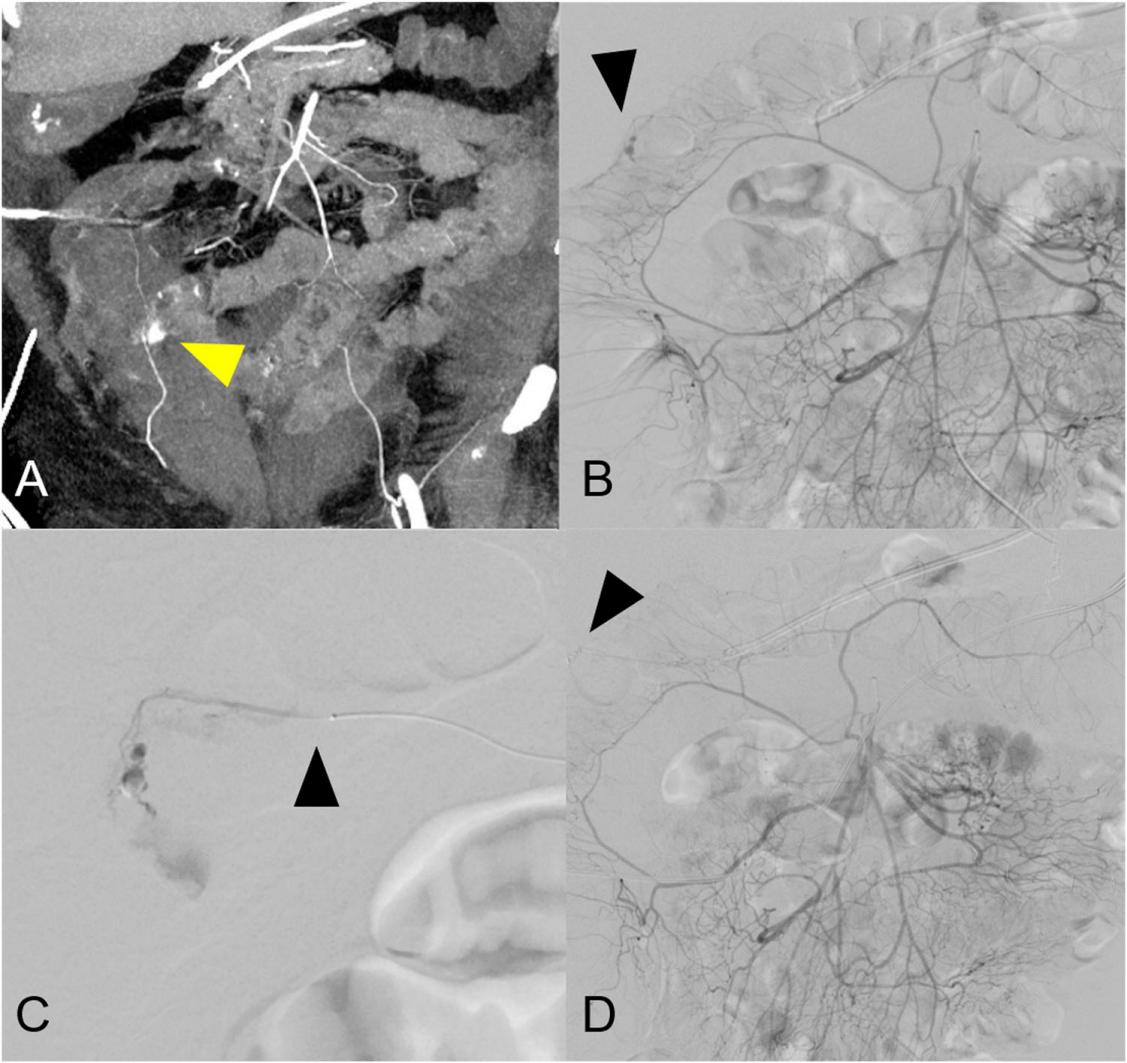
**A** Contrast-enhanced CT in the arterial phase and coronary slice of a patient with massive rectal bleeding following heart surgery and anticoagulation, where endoscopy revealed no abnormalities. The CT scan identified active arterial bleeding in the region of the right colonic flexure (yellow arrowhead). **B** A superior mesenteric angiography revealing extravasation in the area of the right colonic flexure (black arrowhead). **C** A micro-wire and catheter (black arrowhead) were used to navigate into the distal arterial arcade, allowing the bleeding to be successfully treated through coil embolization. **D** The final mesenteric angiogram confirms that the entire remaining bowel is still adequately perfused while the target vessel was closed with a coil (black arrowhead)

**Fig. 4:**
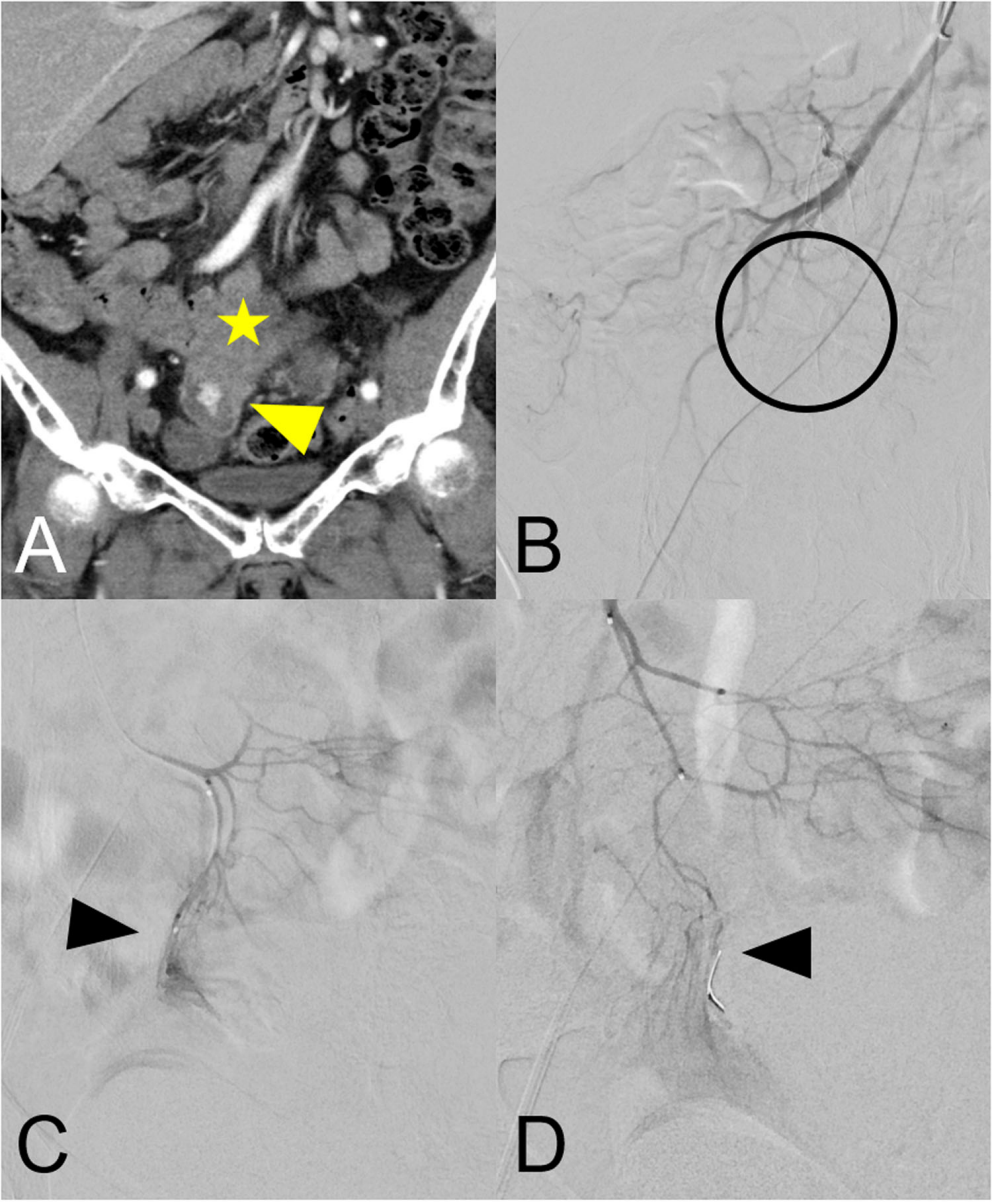
A 73-year-old patient with high-grade serous ovarian carcinoma recurrence and tumor invasion in small bowel loop in small pelvis with Hb-effective bleeding and recurrence in small pelvis. **A** Contrast-enhanced angio-CT in arterial phase and coronary reconstruction shows the tumor adjacent to the bowel loop (yellow star) and active extravasation (yellow arrowhead). **B** No evidence of bleeding on the superior mesenteric angiography from the macrocatheter. After correlation with the angio-CT (**A**) and superselective probing with a microcatheter up to the terminal arcade evidence of bleeding is successful (**C**). After coil embolization, the bleeding stops (**D**)

Since TAE has emerged as a treatment option for patients with GI bleeding, there has been an ongoing debate regarding the most appropriate and safest embolic agent or device. The heated discussion is driven by concerns about potential successive infarctions of intestinal segments, which might necessitate surgical intervention. Given that studies frequently suffer from bias due to being conducted at a single center with specific preferences and expertise for particular embolic agents, our data offer insight into the procedure across five university hospitals in Germany [1, 18]. In our study population, coil embolization was the most frequently used agent, followed by glue and particles. In some patients, different combinations of these three types of embolic agents were used, which is in line with the literature [1]. While coil embolization carries the lowest risk of ischemia, coils provide an opportunity for collateralization and revascularization in cases of severe coagulation restriction or vasopressor use [19–21]. These risks are lower when glue is used, for which there is also a growing body of evidence regarding GI bleeding. Naturally, its application requires more expertise [6]. Regarding particles, there is a consensus against using excessively small ones. A recently published study demonstrates an acceptable safety profile for standard-sized particles. Combining coils and particles is also a promising solution [22–24]. Concerning the risk of infarction, the selectivity of the embolization position seems to be more important than the choice of embolic agents [1]. Overall, it is important to aim for the most selective positioning possible to minimize the risk of ischemia. This is particularly relevant given recent advances in embolization techniques, which allow the materials used to be tailored to treat LGIB effectively. For instance, in genicular artery embolizations, the smallest vessels are treated using wires and catheters are typically employed in neuroradiological interventions [25]. Given that superselective embolization carries the lowest bleeding risk, it is worth considering the use of these materials in this context as well (Figs. 3 and 4).

Intestinal ischemia, along with the subsequent need for surgical resection, poses the most significant concern in TAE. A low risk of approximately 5% has been reported in the literature [1, 26]. At 14%, our data is slightly higher. One reason for this may be the high number of clinically unstable patients who are more likely to develop intestinal ischemia due to their clinical condition (in the context of sepsis or the need for vasoconstrictor medications). In addition, the high rates of technical and clinical success are an indication that stopping the bleeding was a high priority in all centers. In addition, at most of the centers that participated in the study, the majority of patients first underwent endoscopy, and the cohort was selected by treatment failures. Total 30-day mortality was 14.0%, indicating that LGIB is a life-threatening situation that can be treated thanks to standardized guidelines and interdisciplinary treatment strategies. The overall clinical constellation certainly represents the major factor influencing short-term mortality. Bleeding-associated mortality would be of interest, but difficult to determine with respect to the retrospective nature of the analysis. However, due to the high technical and clinical success rate, it can be assumed that this figure is low.

Therefore, our study has some limitations. As mentioned, the retrospective and multicenter design introduces the possibility of variations in patient selection, interventional technique, and the experience level of interventionalists, all potentially affecting treatment outcomes. Due to the retrospective nature of the study, data on individual parameters may be incomplete. Furthermore, inherent variations in the causes of hemorrhage may distort results. It is important to note that the study is purely descriptive in nature and lacks comparison with other techniques.

## Conclusion

While endovascular treatment of LGIB using TAE appears to play an increasingly important role in the care of clinically unstable patients, our multicenter data reveal a high technical and clinical success rate with a risk profile deemed acceptable. However, there remains a risk of subsequent intestinal ischemia with the need for surgery, underscoring the importance of a judicious assessment of indications. Moreover, efforts should be directed toward achieving the most selective embolization position feasible, with embolization conducted cautiously, despite no clear superiority demonstrated for any embolization material in this study. In the end, there is still no head-to-head comparison with endoscopic procedures. Therefore, urgent clarification through prospective controlled studies is necessary to answer this crucial question.

## Supplementary information

ELECTRONIC SUPPLEMENTARY MATERIAL

## Abbreviations

CT: Computed tomography
CTA: Computed tomography angiography
GI: Gastrointestinal
LGIB: Lower gastrointestinal bleeding
TAE: Trans-arterial embolization
