# Supplementary material for: Trans-arterial embolization for treatment of acute lower gastrointestinal bleeding—a multicenter analysis
Source: Eur Radiol. 2024 Oct 16;35(5):2746–54. doi: 10.1007/s00330-024-11102-x (PMC12021941; doi:10.1007/s00330-024-11102-x)
Supplement: Supplementary file 1 — ELECTRONIC SUPPLEMENTARY MATERIAL [file 330_2024_11102_MOESM1_ESM.pdf]

Trans-arterial Embolization for Treatment of Acute Lower Gastrointestinal Bleeding – A Multicenter Analysis

ELECTRONIC SUPPLEMENTARY MATERIAL

Supplement 1:

At **Center 1 (Berlin)**, we typically use a 4-5 F sheath (Radifocus Introducer II; Terumo, Tokyo, Japan) and 4-5 F macrocatheters in Cobra and SIM 1 configurations (Cordis, Hialeah, USA) for non-selective angiography. For microcatheters, we generally use a 2.5 F Cantata (Boston Scientific, Marlborough, USA) or a 2.4 F Terumo Progreat (Terumo, Tokyo, Japan). Microwires typically used include the Fathom 14 or 16 (Boston Scientific, Marlborough, USA) and the Terumo 90° (Terumo, Tokyo, Japan). Permanent embolic material includes pushable coils (Hilal, Tornado, and Nester, Cook, Bloomington, USA). For permanent particulate embolization, we use non-calibrated particles sized 355-510 µm (Boston Scientific, Marlborough, USA), and Gelaspon is used for temporary embolization. For glue embolization, n-butyl cyanoacrylate (Histoacryl, Braun, Kronberg im Taunus, Germany, and Glubran, GEM, Viareggio, Italy) is used in various mixtures with lipiodol (Guerbet, Villepinte, France). For plug embolization, we use the Vascular Amplatz II Plug (Abbott, Chicago, USA).

At **Center 2 (Mainz)**, we typically use a 5 F sheath (AVANTI, Cordis, Hialeah, USA) and 5 F macrocatheters in Cobra and SIM 1 configurations (Radifocus Glidecath, Terumo, Tokyo, Japan) for non-selective angiography. For microcatheters, we generally use a 2.4 F Renegade (Boston Scientific, Marlborough, USA) or a 2.0 F Progreat (Terumo, Tokyo, Japan). Microwires typically used include the Fathom 14 or 16 (Boston Scientific, Marlborough, USA) and the Terumo 90° (Terumo, Tokyo, Japan). Permanent embolic material includes pushable coils (Pushable 0.018" Fibered Platinum Coils in various shapes, Boston Scientific, Marlborough, USA) and detachable coils (Interlock Fibered IDC Occlusion System, Boston Scientific, Marlborough, USA). For permanent particulate embolization, we use non-calibrated particles sized 100-300 µm, 300-500 µm and 500-700µm (Bead Block, Boston Scientific, Marlborough, USA), and Gelaspon is used for temporary embolization. For glue embolization, n-butyl cyanoacrylate (Histoacryl, Braun, Kronberg im Taunus, Germany, and Glubran, GEM, Viareggio, Italy) is used in various mixtures with lipiodol (Guerbet, Villepinte, France). For plug embolization, we use the Vascular Amplatz II Plug (Abbott, Chicago, USA).

At **Center 3 (Hannover)**, we typically use a 4-5 F sheath (Anvanti+; Cordis, Miami Lakes, Florida, USA) and 4-5 F macrocatheters in Cobra and SH 1.0 configurations (Cordis, Hialeah, USA) for non-selective angiography. For microcatheters, we generally use a 2.4 F or 2.0 F Maestro or Persue (Merit Medical, South Jorden, Utah, USA). Microwires typically used include the Fathom 14 or 16 (Boston Scientific, Marlborough, USA) and the TrueForm (Merit Medical, South Jorden, Utah, USA). Permanent embolic material includes pushable coils (Nester, Cook, Bloomington, USA; VortX, Boston Scientific, Marlborough, USA). For permanent particulate embolization, we use non-calibrated particles sized 355-510 µm (Boston Scientific, Marlborough, USA), and Gelaspon is used for temporary embolization. For glue embolization, n-butyl cyanoacrylate (Histoacryl, Braun, Kronberg im Taunus, Germany, and Glubran, GEM, Viareggio, Italy) is used in various mixtures with lipiodol (Guerbet, Villepinte, France). For plug embolization, we use the Vascular Amplatz II Plug (Abbott, Chicago, USA).

At **Center 4 (Lübeck)**, we typically use a 5 F sheath (Radifocus Introducer II; Terumo, Tokyo, Japan) and 5 F macrocatheters in Cobra and SIM 1 configurations (Radifocus Glidecath,

Terumo, Tokyo, Japan) for non-selective angiography. For microcatheters, we generally use a 2.4 F Renegade (Boston Scientific, Marlborough, USA) or a 2.0 F Progreat (Terumo, Tokyo, Japan). Microwires typically used include the Fathom 14 or 16 (Boston Scientific, Marlborough, USA). Permanent embolic material includes pushable coils (Pushable 0.018" Fibered Platinum Coils in various shapes, Boston Scientific, Marlborough, USA) and detachable coils (Interlock Fibered IDC Occlusion System, Boston Scientific, Marlborough, USA and Ruby Coil / POD® System, Alameda, CA, USA). For permanent particulate embolization, we use non-calibrated particles sized 100-300 µm, 300-500 µm and 500-700µm (Embosphere Microspheres, Merit Medical Systems Inc., South Jordan, United States), and Gelaspon is used for temporary embolization. For glue embolization, n-butyl cyanoacrylate (Histoacryl, Braun, Kronberg im Taunus, Germany, and Glubran, GEM, Viareggio, Italy) is used in various mixtures with lipiodol (Guerbet, Villepinte, France). For plug embolization, we use the Vascular Amplatz II Plug (Abbott, Chicago, USA).

At **Center 5 (Essen)**, we typically use a 5 F sheath (Prelude; Merit Medical, Uta, USA) and a 5 F macrocatheters in SIM 1 configuration (Impress, Merit Medical, Uta, USA) for non-selective angiography. For microcatheters, we generally use a 2.4 F Renegade (Boston Scientific, Marlborough, USA) or a 1.98 F Parkway Soft (Asahi Intecc, Asahi, Japan). Microwires typically used include the Runthrough NS Floppy (Terumo, Tokyo, Japan). Permanent embolic material includes pushable coils (Pushable 0.018" Fibered Platinum Coils in various shapes, Boston Scientific, Marlborough, USA) and detachable coils (Interlock Fibered IDC Occlusion System, Boston Scientific, Marlborough, USA). For permanent particulate embolization, we use non-calibrated particles sized 150-250 µm, 250-355 µm and 500-710µm (Contour, Boston Scientific, Marlborough, USA), and Gelaspon is used for temporary embolization. For glue embolization, n-butyl cyanoacrylate (Histoacryl, Braun, Kronberg im Taunus, Germany, and Glubran, GEM, Viareggio, Italy) is used in various mixtures with lipiodol (Guerbet, Villepinte, France). For plug embolization, we use the Vascular Amplatz II & IV Plug (Abbott, Chicago, USA).

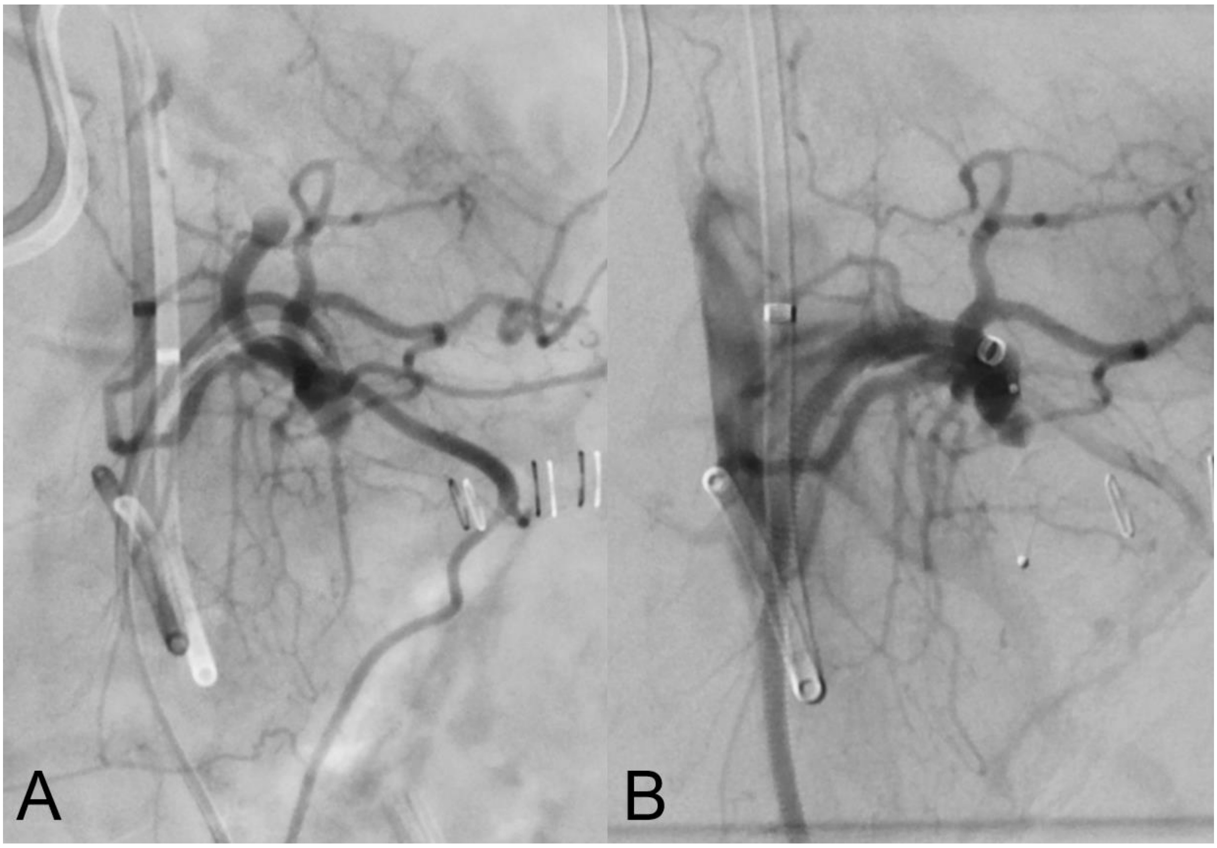

Figure Supplement 1: *A 42-year-old patient experienced a postoperative complication following pancreatic surgery, resulting in injury to a jejunal loop and the distal SMA. After a hemorrhage from a vessel associated with a jejunal arcade (A), successful plug embolization was performed (B).*

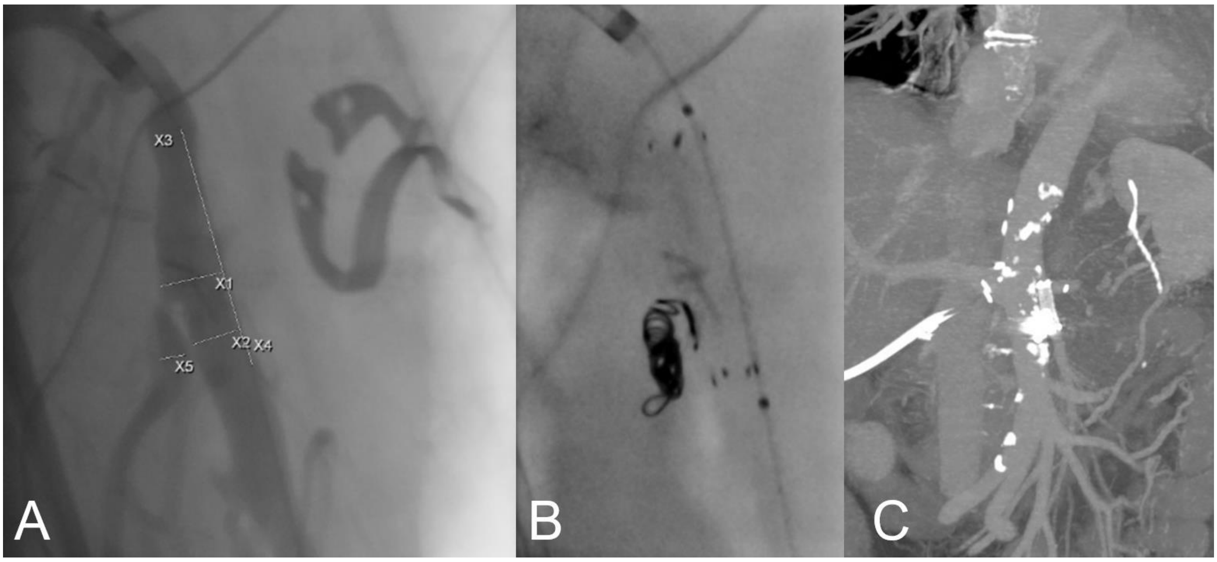

Figure Supplement 2: A patient presents with a pseudoaneurysm near a jejunal arcade following Whipple surgery for pancreatic carcinoma, complicated by intraluminal blood leaks. The proposed approach includes: A) Planning angiography, B) A combined procedure of coil embolization and stent graft implantation, and C) Post-interventional CT imaging.

Eur Radiol (2024) Hosse C, Moos M, Becker LS, et al.
